# Incidence, kinetics, and risk factors for intra- and extracranial cerebral arteriopathies in a newborn sickle cell disease cohort early assessed by transcranial and cervical color Doppler ultrasound

**DOI:** 10.3389/fneur.2022.846596

**Published:** 2022-09-14

**Authors:** Françoise Bernaudin, Cécile Arnaud, Annie Kamdem, Isabelle Hau, Fouad Madhi, Camille Jung, Ralph Epaud, Suzanne Verlhac

**Affiliations:** ^1^Department of Pediatrics, Referral Center for Sickle Cell Disease, Intercommunal Créteil Hospital, University Paris Est, Créteil, France; ^2^Clinical Research Center, Intercommunal Créteil Hospital, University Paris Est, Créteil, France; ^3^Department of Pediatrics, Intercommunal Créteil Hospital, University Paris Est, Créteil, France; ^4^Department of Medical Imaging, Referral Center for Sickle Cell Disease, Intercommunal Créteil Hospital, Créteil, France

**Keywords:** sickle cell disease/anemia, transcranial and cervical color-Doppler, ultrasound, cerebral MRI/MRA, cerebral arterial stenosis, neck-MRA, hydroxyurea, silent cerebral infarct

## Abstract

The risk of stroke in children with sickle cell disease (SCD) is detected by abnormal intracranial arterial time-averaged mean of maximum velocities (TAMVs ≥200 cm/s). Recently, extracranial internal carotid artery (eICA) arteriopathy has been reported, and a cross-sectional study showed that eICA-TAMVs ≥160 cm/s are significantly associated with eICA kinkings and stenosis. The cumulative incidence of and predictive risk factors for intracranial arteriopathy are well described in sickle cell anemia (SCA=SS/Sβ0) but are lacking for SC/Sβ+ children, as is the cumulative incidence of eICA arteriopathy. We report a prospective longitudinal cohort study including 493 children with SCD (398 SCA, 95 SC/Sβ+), all assessed by transcranial and cervical color Doppler ultrasound. Cerebral MRI/MRA data were available in 375 children with SCD and neck MRA in 365 children. eICA kinkings were defined as eICA tortuosities on neck MRA, with an internal acute angle between the two adjacent segments <90°. The median follow-up was 10.6 years. The cumulative incidence of kinkings was significantly lower in SC/Sβ+ children than in children with SCA, and no SC/Sβ+ child developed intra- or extracranial stenotic arteriopathy. The 10-year KM estimate of cumulative incidence (95% CI) for eICA-TAMVs ≥160 cm/s revealed its development in the 2nd year of life in children with SCA, reaching a plateau of 17.4% (13.2–21.6%) by about 10 years of age, while the plateau for eICA stenosis was 12.3% (8.3–16.3%). eICA assessment identified 13.5% (9.3–17.7%) patients at risk of stroke who were not detected by transcranial color Doppler ultrasound. We also show, for the first time, that in addition to a congenital origin, eICA kinkings sin patients with SCD can develop progressively with aging as a function of eICA-TAMVs, themselves related to anemia severity. Ongoing hydroxyurea treatment was significantly associated with a lower risk of abnormal intracranial arteriopathy and eICA kinkings. After adjustment with hydroxyurea, baseline low hemoglobin, high reticulocyte, and WBC counts remained independent risk factors for intracranial arteriopathy, while low hemoglobin and SEN β-haplotype number were independent risk factors for extracranial arteriopathy. The association between extracranial arteriopathy and SEN β-haplotype number suggested a genetic link between the ethnic origin and incidence of eICA kinkings. This prospective cohort study shows the importance of systematically assessing the eICA and of recording biological parameters during the 2nd year of life before any intensive therapy to predict the risk of cerebral arteriopathy and treat patients with severe baseline anemia.

## Introduction

The cumulative incidence of and predictive risk factors for intracranial arteriopathy have been well described in children with sickle cell anemia (SCA; i.e., SS, Sβ0, and SDPunjab) but are lacking for SC/Sβ+ children. Strokes are most often associated with stenosis of the large arteries of the intracranial anterior circulation (1). Transcranial Doppler (TCD) screening assesses time-averaged mean of maximum velocities (TAMVs) in the middle cerebral artery (MCA), anterior cerebral artery (ACA), and internal carotid artery (ICA) (2, 3). Abnormal high TAMVs (≥200 cm/s) identify patients with a 40% stroke risk within 36 months (4) and are associated with a risk of silent cerebral infarcts (SCI) (5–7). In 1998, prophylactic red cell chronic transfusion programs were shown to significantly reduce the incidence of strokes among patients at risk (STOP-I) (8).

While the involvement of the circle of Willis is certainly predominant in SCA cerebral vasculopathy, less attention has been given to the extracranial internal carotid artery (eICA), which can be the site of stenosis and/or occlusion and is also responsible for overt strokes and SCI (9–13). Contrary to intracranial arteriopathy detectable by TCD *via* a temporal window and by cerebral MRA, assessing the eICA requires using a color Doppler ultrasound through a submandibular approach (11) and neck MRA (9–14). In two large cohorts of stroke-free children with SCA, we reported in a cross-sectional study (14) that eICA-TAMVs ≥160 cm/s are highly associated with kinkings and eICA stenosis (14), which were independent risk factors for SCI, along with acute and chronic anemia (7).

Since this first description, there has been no report on the cumulative incidence of extracranial cerebral arteriopathy during childhood and the associated predictive risk factors. The aim of the present study was to evaluate the cumulative incidence of and predictive risk factors for extracranial cerebral arteriopathy and to compare them with cumulative incidence of and predictive risk factors for intracranial arteriopathy in a newborn SCD (SCA & SC/Sβ+) cohort, longitudinally assessed for cerebral intra- and extracranial arteriopathies.

## Methods and patients

Since 1992, our center has systematically assessed patients as soon as 18 months of age by using a transcranial color Doppler ultrasound machine (14). The assessment of the eICA has been performed *via* submandibular windows using the same low-frequency probe as for the TCD since June 2011 (14). Starting in May 1993, MRI/MRA with a 1.5T magnet with FLAIR, T1, T2, SWI, diffusion-weighted sequences, circle of Willis, and 3D time-of-flight (TOF) angiography were performed without sedation every 2 years in children older than 5 years, but earlier in patients on chronic transfusion for abnormal TCD and systematically before hematopoietic stem cell transplantation. Neck 3D TOF angiography (neck MRA) was added in June 2011 (14). Neck MRA was systematically performed in children older than 5 years but earlier in those with abnormally high eICA velocities. All imaging data were reviewed by the same expert (SV). Arteries were assessed for shape deformations and stenosis, defined as at least a 25% decrease in the lumen based on diameter. Tortuosities can be classified into three types, namely, loop, coiling, and kinking (15, 16). Loops are defined as S- or C-shaped deformities coiling as a circular course, while kinking is described as a sharp angulation. For the present study, only kinkings with an internal acute angle between the two adjacent segments <90° were retained for analysis. Stenosis was defined as a 25% decrease at least in the lumen of MCA, ICA, ACA, or eICA.

The present cohort included children born between Jan 1988 and Jan 2018 who were followed in the center at least until June 2012 in order to systematically assess annually by TCD and at least once by cervical color Doppler ultrasound. Parental written informed consent was obtained in accordance with the Declaration of Helsinki, and data were prospectively and systematically recorded in the clinical database of the referral center for SCD in Créteil (CNIL, N° 2069568). The use of the database was approved for this cohort study by the Créteil Institutional Review Board.

Indications for intensive therapy were as follows: Hydroxyurea has been prescribed since 1992, initially in patients older than 3 years and experiencing frequent vaso-occlusive crises (VOC) and/or acute chest syndrome (ACS) (17). Since 1998, a subset of patients with an abnormal TCD history but with normalized velocities on chronic transfusion and no stenosis on MRA have been prescribed hydroxyurea (18, 19). Moreover, because of the proven negative effect of anemia on cognitive performance (20), hydroxyurea has also been given to patients with normal TCD but hemoglobin <7 g/dl since 2000. Thereafter, hydroxyurea was also recommended to symptomatic children during the 2nd year of life after their first complete checkup including TCD. More recently, considering the safety and efficacy of the Baby HUG trial (21) and the NIH recommendations (22), hydroxyurea was occasionally prescribed as soon as 9 months of age. Chronic transfusion was recommended in children experiencing at least two acute splenic sequestrations until the recommended age for splenectomy, in those with intracranial or eICA-TAMVs ≥200 cm/s or stenosis on cerebral or neck MRA and in those still experiencing frequent VOC/ACS on hydroxyurea. Transplantation was recommended to patients with a matched sibling donor (MSD) who experienced frequent VOC/ACS despite hydroxyurea or to those with cerebral vasculopathy defined by the presence of intra- or extracranial stenotic arteriopathy, abnormal TAMV, or the presence of ischemic lesions.

The follow-up was from Jan 1988 to Sept 2019. Alpha-genes, beta-globin haplotypes (BEN, CAR, SEN, and others), and G6PD enzymatic activity were recorded. Average biologic parameters were obtained at baseline after the age of 12 months and before the age of 3 years, a minimum of 3 months away from a transfusion, 1 month from a painful episode, and before any intensive therapy.

## Statistical analysis

Participant baseline characteristics were summarized through the use of percentages, and mean (standard deviation, SD) or median (range); 95% confidence intervals (95% CIs) around point estimates were computed. Fisher exact tests were used to compare proportions and Mann–Whitney tests to compare continuous distributions.

Birth date defined entry into the study. For Kaplan–Meier estimates of cumulative incidence (probability) of events, the participants were censored on the date of last visit or event, that is, TAMVs ≥200 cm/s for intracranial arteries and ≥160 cm/s for the eICA, or stenosis on cerebral and neck MRA. Failure time data curves were compared across baseline groups by the log-rank test. Because only six deaths occurred among 493 patients, they were not considered competing risks for studied events but treated as noninformative censoring observations at the time of death, similar to children who had not experienced any event of interest and were censored at the time of their last visit. The presence or not of ongoing treatment by hydroxyurea for at least 6 months was recorded for each event in each patient.

Association between outcomes and baseline variables was assessed using Cox regression with the estimated hazards ratio (HR) and 95% CI and adjusted with ongoing hydroxyurea treatment. The absence of violation of the proportional hazard assumption was checked in the analyses. Univariable models were fitted, and variables associated with the outcome at the 20% level were retained for introduction into a multivariable model, except for those with strong correlation such as hematocrit with hemoglobin and neutrophils with the WBC count. Multivariate analyses used a stepwise selection process that consists of a series of alternating forward selection and backward elimination steps. All statistical tests were two-sided, with p-values of 0.05 or less denoting statistical significance.

Statistical analyses were performed with SPSS version 24, R version 4.0.2, and MedCalc (Belgium) software packages.

## Results

This study included 493 children (238 F, 255 M) with SCD [398 SCA (385 SS, 10 Sβ0, and three SDPunjab), 65 SC, and 30 Sβ+ patients], all assessed by TCD before 2 years of age and at least once with cervical Doppler. Those born after June 2009 (*N* = 205) were simultaneously assessed by intracranial and cervical Doppler before 2 years of age.

In the overall cohort, the median (range) follow-up was 10.6 (1.1–22.9) years, providing 5,335 patient-years of follow-up. A total of three children with SCA had an ischemic stroke: the first one had an abnormal TCD at 18 months of age (235 cm/s on left MCA) and had a stroke 1 month later, just before the confirmatory TCD; the second one had normal left-sided velocities but no available temporal window on the right side and had a stroke related to severe right MCA stenosis at 4.4 years of age; the third child had normal TCD but developed a febrile ACS at age 4.2 years and experienced a massive bilateral MCA and ACA thrombosis and died. Overall, five deaths occurred in children with SCA: the first one at age 4.2 years was related to the overt stroke described before, two others were related to sepsis at ages 2 and 7 years, and two occurred after transition at age 19 years related to complicated haplo-identical transplant and at age 20 years related to severe ACS; one death occurred in an Sβ+ patient at age 13 years during the course of ACS related to acute pulmonary hypertension with thrombosis of the inferior vena cava. Hydroxyurea was prescribed in 235/398 (59.0%) patients with SCA at the median age of 5.1 (0.7–17.8) years and in 3/95 (3.2%) Sβ+ patients at 3, 4, and 6 years. Indications for hydroxyurea were frequent VOC/ACS (*N* = 132), baseline hemoglobin lower than 7 g/dL (*N* = 28), a history of TAMV ≥ 200 cm/s after normalization on chronic transfusion, and no stenosis (*N* = 75). Chronic transfusion was started in 221 SCA children at a median age of 3.6 (0.5–17.1) years and in two Sβ+ children. Indications for chronic transfusion in children with SCA were abnormal TAMVs ≥ 200 cm/s (*N* = 116) in intracranial (*N* = 97) or in extracranial arteries (*N* = 19), intracranial stenosis or eICA stenosis not associated with abnormal TAMVs ≥ 200 cm/s (*N* = 25), and recurrent splenic sequestrations or frequent VOC/ACS despite hydroxyurea in the others. The patients with a history of abnormal TAMV placed on chronic transfusion who were switched to hydroxyurea (*N* = 75) received both until the maximal tolerated dose of hydroxyurea was reached. Stem cell transplantation was performed in 70 patients with SCA from a matched sibling donor (*N* = 66) or haploidentical donor (*N* = 4) at a median age of 6.7 (3.2–19.7) years. The main indication for transplantation was the presence of cerebral vasculopathy (*N* = 40): history of overt stroke (*N* = 1), intra- or extracranial arteriopathy on MRA (*N* = 24), abnormal TAMV without stenosis (*N* = 11), presence of silent cerebral infarcts without stenotic arteriopathy but associated with other complications (N = 6), or hydroxyurea failure to decrease the rate of VOC/ACS or to avoid other complications (*N* = 30).

Among the 493 patients with SCD, G6PD activity was available in 439 patients, and deficit was present in 63 of them (13.9%). Alpha genes were available in 454 patients, and 167 (36.8%) had a deletion of at least one α-gene. Beta-haplotypes were available in 417 patients, and 268 of them were homozygous: CAR/CAR (*N* = 131), BEN/BEN (*N* = 91), and SEN/SEN (*N* = 46), while the others were heterozygous. Baseline mean (SD) biological parameters of patients with SCA and SC/Sβ+ patients and in patients with CAR/CAR, BEN/BEN, and SEN/SEN are shown (Table 1). Proportions of patients with intra- or extracranial arteriopathy according to genetic markers and mean (SD) biological parameters in each type of arteriopathy are shown in Table 2.

**Table 1:** Baseline biological parameters in SCD patients, according to genotype (SCA vs. SC/Sb+) and beta-haplotype categories.

	**Genotype**			**Beta-Haplotypes**			
**Biological parameters**	**SS/Sb0**	**SC/Sb+**	***P*-value**	**CAR/CAR**	**BEN/BEN**	**SEN/SEN**	**Other**
Hemoglobin level (g/dL)	8.4 ± 1.3	10.4 ± 0.9	* <0.001*	8.0 ± 1.1	8.6 ± 1.3	9.0 ± 1.6	9.2 ± 1.4
Hematocrit (%)	24.8 ± 4.0	29.9 ± 3.1	<0.001	23.8 ± 3.5	25.4 ± 3.9	25.9 ± 5.9	26.7 ± 3.7
Reticulocyte count (10^9^/L)	292 ± 116	129 ± 63	* <0.001*	297 ± 104	276± 113	275 ± 120	234 ± 138
WBC count (10^9^/L)	13.9 ± 5.0	8.8 ± 2.9	* <0.001*	14.0 ± 5.2	13.7 ± 4.8	13.1 ± 5.1	12.0 ± 5.0
Neutrophil count (10^9^/L)	5.3 ± 2.9	3.4 ± 1.5	* <0.001*	5.5 ± 3.2	5.3 ± 3.0	4.6 ± 2.0	4.6 ± 2.5
Platelet count (10^9^/L)	341 ± 116	321 ± 99	*NS*	340 ± 120	356 ± 122	329 ± 83	328 ± 108
MCV (fL)	76.0 ± 9.3	66.0 ± 6.4	* <0.001*	74.8 ± 11.0	77.0 ± 7.8	78.3 ± 6.7	71.8 ± 9.4
Bilirubin (micromol/L)	29.7 ± 16.2	13.2 ± 5.1	* <0.001*	31.9 ± 15.3	28.2 ± 20.0	29.6 ± 16.5	21.4 ± 12.9
LDH (IU/L)	710 ± 342	394 ± 151	* <0.001*	828 ± 406	663 ± 282	558 ± 233	549 ± 292
HbF (%)	17.1 ± 7.9	9.0 ± 7.8	* <0.001*	14.1 ± 6.7	18.7 ± 8.2	21.7 ± 7.8	12.1 ± 8.3

Average biologic parameters were obtained at baseline after the age of 12 months and before the age of 3 years, a minimum of 3 months away from a transfusion, 1 month from a painful episode, and before any intensivetherapy.

Except platelet count, which is similar in SCA and SC/Sb+ children, all other parameters are highly significantly (*p* < 0.001) different in both populations, that is, in SCA compared to SC/Sb+ children, hemoglobin and hematocrit were lower, while WBC, neutrophils, reticulocyte counts, MCV, bilirubin, LDH, and HbF% werehigher.

Hemoglobin and hematocrit were significantly lower in patients with CAR/CAR than in those with BEN/BEN (*p* = 0.001) and (*p* = 0.005), respectively, and in patients with SEN/SEN (*p* = 0.001) and (*p* = 0.010), respectively, but were not different between BEN/BEN and SEN/SENpatients.

Hemoglobin F% was significantly lower in patients with CAR/CAR than in patients with BEN/BEN (*p* < 0.001) and SEN/SEN (*p* < 0.001) and was lower in patients with BEN/BEN than in those with SEN/SEN patients but not significantly (*p* =0.054).

LDH was significantly higher in patients with CAR/CAR than in those with BEN/BEN (*p* = 0.004) and in those with SEN/SEN (*p* < 0.001) and was higher in patients with BEN/BEN than in those with SEN/SEN but not significantly (*p* =0.051).

Thus, patients with CAR/CAR were the most anemic with the most hemolysis and with the lowest HbF%, while patients with SEN/SEN were the less anemic with the least hemolysis and had the highestHbF%.

**Table 2:** Proportions of patients with SCD with intra- and extracranial arteriopathies according to genetic markers, baseline biologic parameters, and ongoing hydroxyurea treatment for at least 6 months at each event.

	**Overall SCD population: SCA and SC/Sb**+ **(*****N*** = **493)**						
		**Proportions**					
		**Intracranial**	**eICA**	**eICA**	**eICA**	**No intra 200&**
		**TAMV≥200**	**Stenosis**	**TAMV≥160**	**Tortuosities**	**Stenosis**	**No eICA≥160**
		**97/493 (19.7%)**	**35/375 (9.3%)**	**62/493 (12.6%)**	**100/365 (30.6%)**	**38/365 (10.4%)**	**348/493 (70.6%)**
**Genetic markers**	***N***						
Genotype (*N* = 493)							
SCA	398	97/398 (24.4%)	10.9%	15.3%	33.5%	11.8%	63.8%
SC/Sb+	95	0/95 (0%)	0/39 (0%)	1/95 (1.1%)	11.6%	0%	98.9%
Gender (*N* = 493)							
M	255	18.4%	8.6%	12.5%	33.3%	12.2%	72.9%
F	238	21%	10.1%	12.6%	27.9%	8.7%	68.1%
G6PD activity (*N* = 439)							
normal	376	18.9%	7.0%	12.5%	30.2%	8.6%	71.3%
deficient	63	28.6%	15.6%	9.5%	26.3%	14.0%	65.1%
Alpha-Thalassemia (*N* = 454)							
absent	287	21.6%	10.8%	13.6%	28.6%	11.1%	68.6%
present	167	18.0%	5.0%	12.6%	33.1%	9.6%	71.3%
Beta Haplotype (*N* = 417)							
CAR/CAR	131	28.2%	11.4%	16.8%	30.4%	12.3%	58.0%
BEN/BEN	91	26.4%	10.4%	13.2%	32.3%	10.4%	64.8%
SEN/SEN	46	13%	2.6%	23.9%	45.9%	17.9%	69.6%
Other	149	16.7%	9.4%	9.3%	26.2%	6.3%	76.0%
**Biological parameters**	**mean** **±SD**						
Hemoglobin level (g/dL)	428	7.8 ± 1.2	7.6 ± 1.0	8.2 ± 1.4	8.3 ± 1.3	8.1 ± 1.2	9.2 ± 1.4
Hematocrit (%)	427	23.4 ± 3.5	21.7 ± 5.5	24.2 ± 4.0	24.6 ± 3.7	24.4 ± 3.7	26.9 ± 4.3
Reticulocyte count (10^9^/L)	419	345 ± 134	369 ± 131	277 ± 101	284 ± 105	293 ± 99	230 ± 119
WBC count (10^9^/L)	428	16.4 ± 5.4	17.7 ± 6.6	13.6 ± 3.9	13.7 ± 4.4	13.7 ± 5.1	11.7 ± 4.7
Neutrophil count (10^9^/L)	419	6.3 ± 3.3	6.5 ± 4.0	5.4 ± 2.6	5.0 ± 2.4	4.9 ± 2.8	4.5 ± 2.5
Platelet count (10^9^/L)	426	336 ± 130	342 ± 102	314 ± 105	337 ± 112	318 ± 104	337 ± 110
MCV (fL)	424	79.0 ± 7.4	81.9 ± 6.2	76.4 ± 7.7	75.6 ± 7.9	76.7 ± 8.3	71.9 ± 10.0
Bilirubin (mmol/L)	334	34.8 ± 17.7	30.3 ± 14.8	29.9 ± 13.5	29.5 ± 14.4	27.6 ± 10.9	23.0 ± 15.2
LDH (IU/L)	372	858 ± 385	1026 ± 412	662 ± 312	657 ± 313	675 ± 327	586 ± 311
HbF (%)	416	14.2 ± 6.4	14.5 ± 5.5	16.4 ± 8.4	16.5 ± 8.0	16.7 ± 7.2	15.5 ± 9.0
	**Proportions**						
Hemoglobin <7g/dL	40	21/40: 52.5%	7/38: 18.4%	11/40: 27.5%	12/35: 34.3%	5/35: 14.3%	11/40: 27.5%
WBC count > 20 x 109/L	38	20/38: 52.6%	8/34: 23.5%	5/38: 13.2%	9/32: 28.1%	5/33: 15.1%	15/38: 39.5%
Reticulocyte count > 400 x 10^9^/L	54	21/54: 38.9%	8/45: 17.8%	6/54: 11.1%	13/41: 31.7%	4/43: 9.3%	28/54: 51.9%
	**Ongoing hydroxyurea treatment at each event**						
	*N*	143/493	102/375	216/493	169/365	206/365	NA
Event on ongoing HU treatment		12/143: 8.4%	11/102: 5.4%	22/216: 10.2%	36/169: 21.3%	13/206: 6.3%	NA

**Figure 1:**
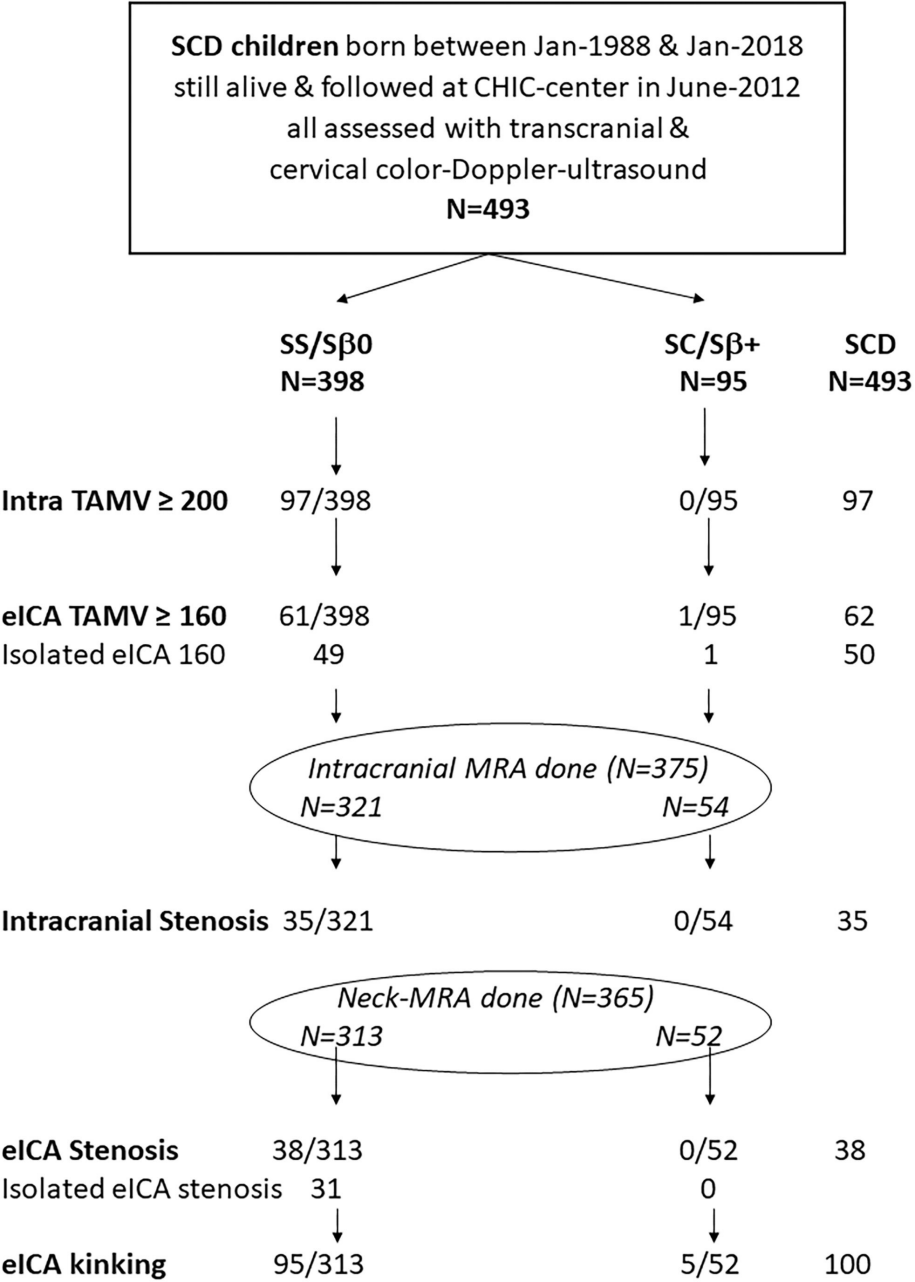
Flowchart of the newborn SCD cohort study systematically assessed by transcranial and cervical color Doppler ultrasound.

The flowchart of the study is presented in Figure 1.

## Comparative cumulative incidence (95% CI) of intra- and extracranial arteriopathies in children with SCD

All data were calculated at 10 years of age (median follow-up).

### Intracranial arteriopathy

#### Intracranial TAMVs ≥200 cm/s

**Figure 2:**
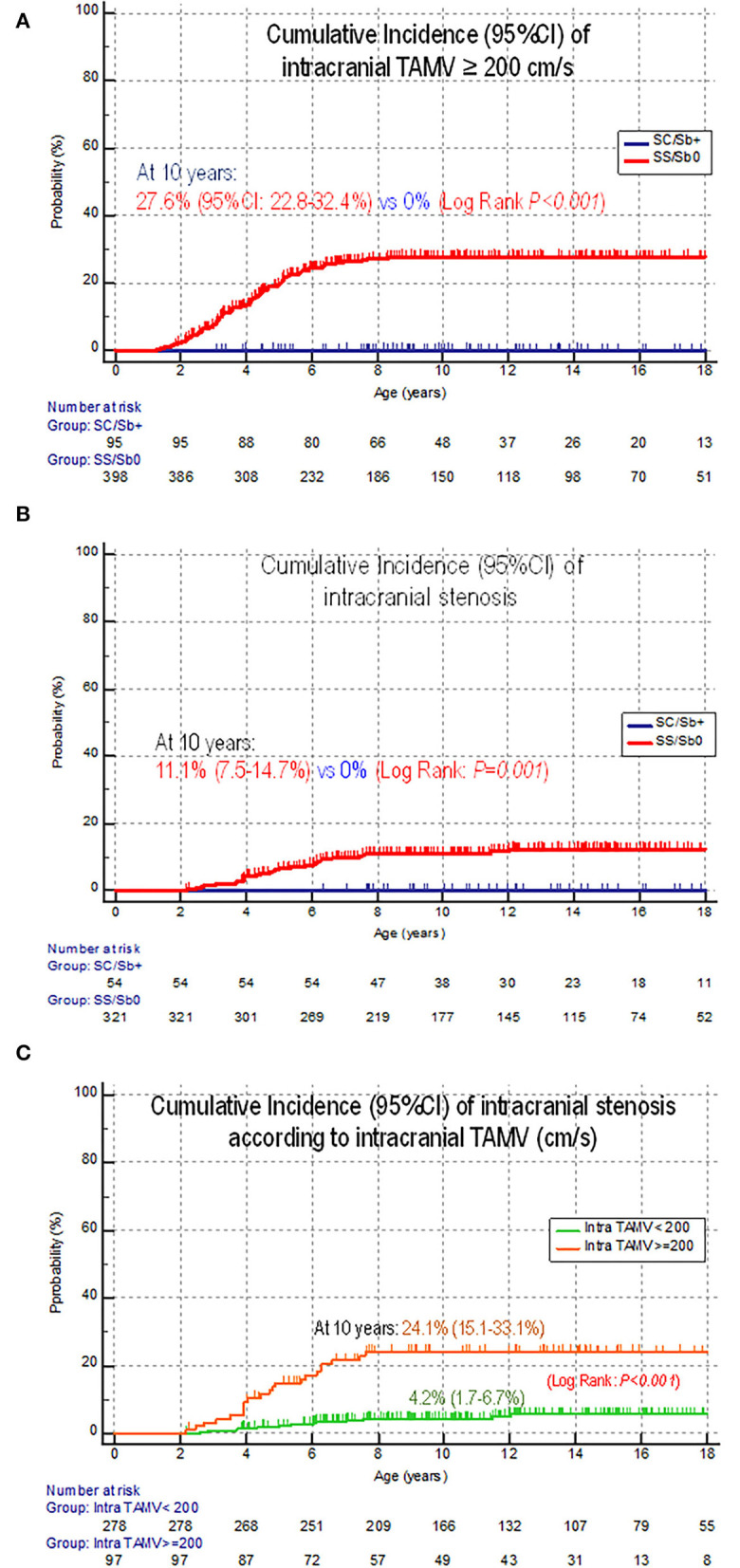
Comparative cumulative incidence of intra- and extracranial arteriopathies in SCA vs SC/Sb+ children and according to TAMV. **(A)** Intracranial TAMV ≥ 200 cm/s. **(B)** Intracranial stenosis. **(C)** Intracranial stenosis according to a history of TAMV (< or ≥ 200 cm/s). Of note, among the 13 patients with stenosis but no history of TAMVs ≥200 cm/s, 10 had a history of conditional TAMV (170–199 cm/s), one had no temporal window with underlying severe intracranial arteriopathy, one had a stroke related to extracranial arteriopathy, and no obvious reason was found in one patient.

TAMVs ≥200 cm/s were observed in 97 children with SCA at a median age of 3.8 (1.3–8.7) years. The cumulative incidence of intracranial TAMVs ≥200 cm/s was 27.6% (22.8–32.4%) in children with SCA, reaching a plateau by 9–10 years of age, while no SC/Sβ+ child developed abnormal intracranial TAMV (log rank; *p* < 0.001) (Figure 2A).

#### Intracranial stenosis

Intracranial MRA was available in 375 patients with SCD (321 SCA, 54 SC/Sβ+). Intracranial stenosis by MRA was observed only in 35 children with SCA at the median age of 4.8 (2.2–12.0) years. The cumulative incidence of intracranial stenosis was 11.1% (7.5–14.7%) in children with SCA, while no SC/Sβ+ child developed intracranial stenosis (log rank: *p* = 0.001) (Figure 2B). It was significantly higher in those with a history of intracranial TAMVs ≥200 cm/s (*N* = 97): 24.1% (15.1–33.1%) than in the others (*N* = 278): 4.2% (1.7–6.6%) (log rank; *p* < 0.001) (Figure 2C).

### Extracranial arteriopathy

#### eICA-TAMVs ≥160 cm/s

**Figure 3:**
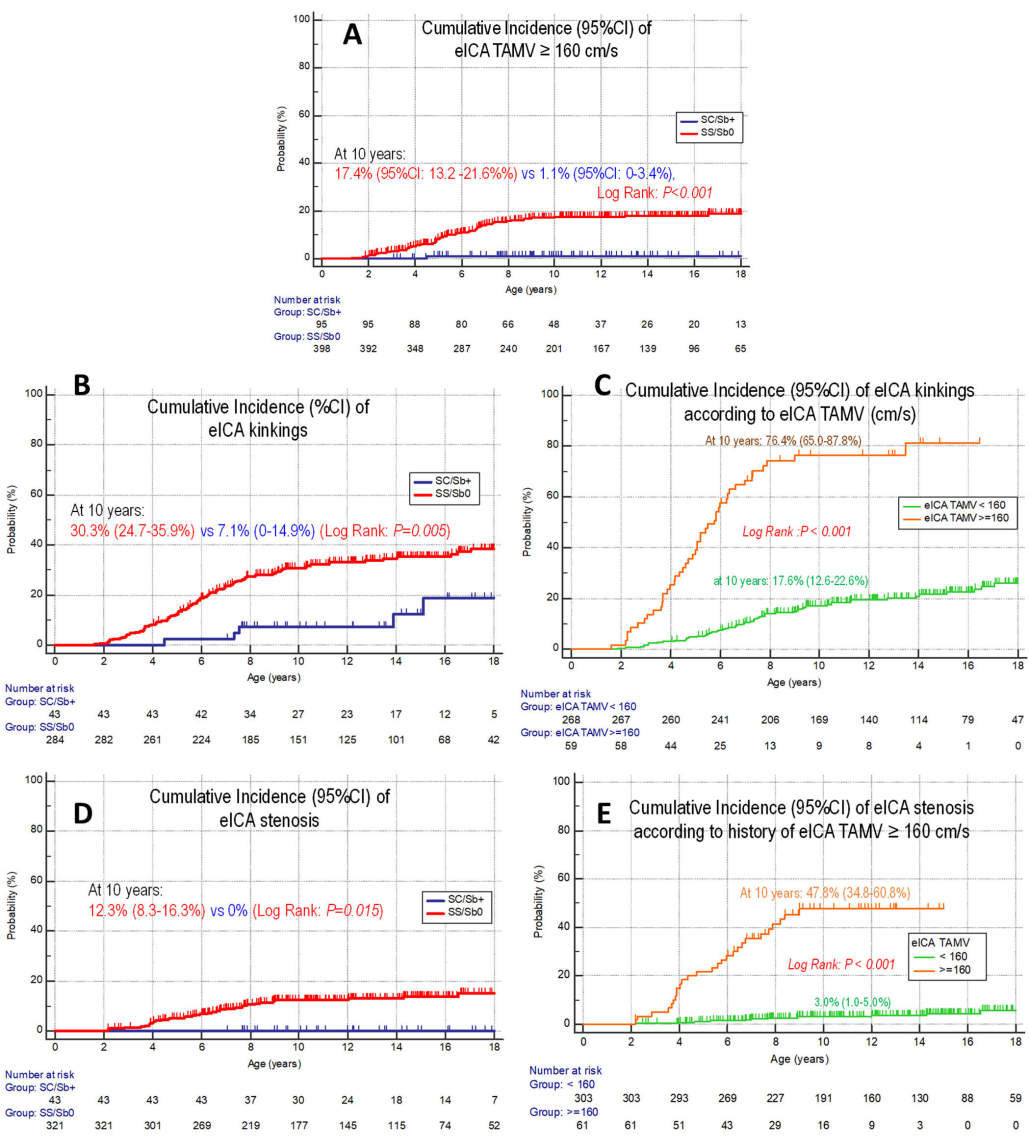
Comparative cumulative incidence of eICA arteriopathy in SCA vs SC/Sb+ children and according to TAMV. **(A)** eICA ≥ 160 cm/s. **(B)** eICA kinkings. **(C)** eICA kinkings according to eICA TAMV (< or ≥ 160 cm/s). **(D)** eICA stenosis. **(E)** eICA stenosis according to eICA TAMV (< or ≥ 160 cm/s).

eICA-TAMVs ≥160 cm/s were observed in 61 of 398 children with SCA and temporarily in one of 95 SC/Sβ+ child. eICA-TAMVs ≥160 cm/s were isolated (without intracranial TAMVs ≥200 cm/s) in 50 of 62 children with SCD. Among the 205 patients with SCD simultaneously assessed with transcranial and cervical color Doppler ultrasound since the 2nd year of life, eICA-TAMVs ≥160 cm/s were observed at the median age of 3.6 (1.3–9.5) years. The cumulative incidence of eICA-TAMVs ≥160 cm/s was 17.4% (13.2–21.6%) in children with SCA and 1.1% (0–3.4%) in SC/Sβ+ children (log rank; *p* < 0.001) (Figure 3A).

In children with SCA, the cumulative incidence of isolated (without intracranial TAMVs ≥200 cm/s) eICA-TAMVs ≥160 cm/s was 14.4% (12.6–16.2%), while that of isolated eICA-TAMVs ≥200 cm/s was 6.2% (3.2–9.2%).

#### eICA kinkings

eICA kinkings were identified by neck MRA in 95 children with SCA and only in five SC/Sβ+ children. Kinkings were present at the first neck MRA in the majority of children but developed secondarily in 12 SCA- and 1 SC/Sβ+ children. The cumulative incidence of eICA kinkings was 30.3% (24.7–35.9%) in children with SCA and 7.1% (0–14.9%) in SC/Sβ+ patients (log rank; *p* = 0.005) (Figure 3B). In children with a history of eICA-TAMVs ≥160 cm/s, the probability of kinkings was 76.4% (64.9–87.9%) vs 17.1% (12.4-21.8%) in those with no history of eICA-TAMVs ≥160 cm/s (log rank; *p* <0.001) (Figure 3C).

#### eICA stenosis

Neck MRA was available in 365 children with SCD. The first neck MRA was performed at the median age of 5.0 (1.6–8.6) years in children born after June 2009. eICA stenosis was present in 38 and isolated (without intracranial stenosis) in 31 children with SCD. All patients with eICA stenosis were children with SCA and not SC/Sβ+ children. The cumulative incidence of eICA stenosis was 13.0% (8.6–17.4%) in children with SCA and 0% in SC/ Sβ+ children (log rank; *p* = 0.015) (Figure 3D) and was 47.8% (34.8–60.8%) in children with a history of eICA-TAMVs ≥ 160 cm/s, but only 3% (0.9–5.1%) in those with eICA-TAMVs <160 cm/s (log rank; *p* < 0.001) (Figure 3E). The cumulative incidence of isolated eICA stenosis was 11.1% (7.3–14.9%).

Of note, chronic transfusion was also required in 13.5% (9.3–17.7%) additional children who had no intracranial arteriopathy but had eICA-TAMVs ≥200 cm/s or eICA stenosis.

## Impact of ongoing hydroxyurea treatment at event

**Figure 4:**
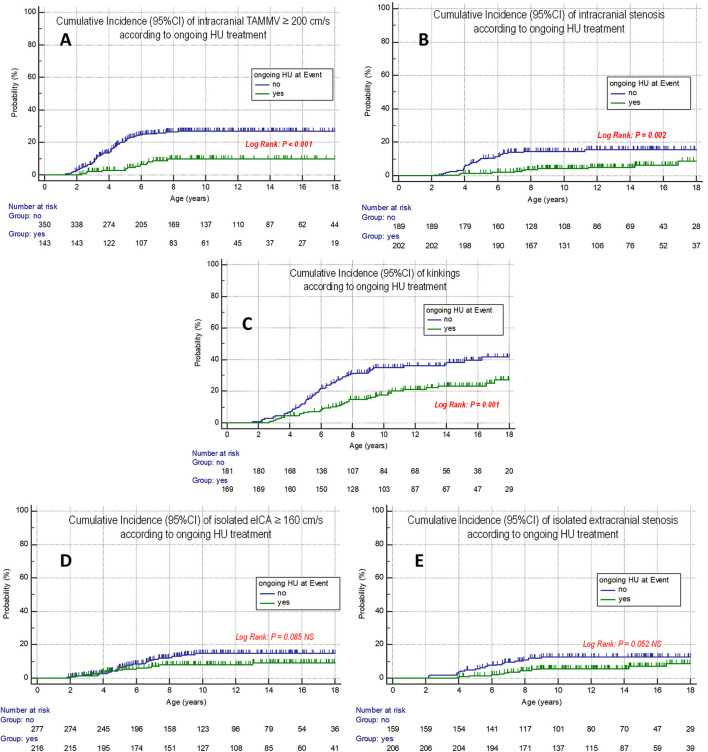
Cumulative incidence of intra- and extracranial arteriopathies in SCD children according to ongoing hydroxyurea treatment at each event. **(A)** Intracranial TAMV ≥ 200 cm/s. **(B)** Intracranial stenosis. **(C)** eICA kinking. **(D)** eICA-TAMV ≥ 160 cm/s. **(E)** eICA stenosis.

Ongoing hydroxyurea treatment at event (Figure 4) was significantly associated with a lower risk of intracranial TAMV ≥ 200 cm/s (log rank; *p* <0.001), intracranial stenosis (log rank; *p* = 0.002), and eICA kinkings (log rank; *p* = 0.001) and a trend to a lower risk of eICA-TAMV 160 cm/s and eICA stenosis.

## Predictive risk factors for intra- and extracranial arteriopathies

Considering the impact of hydroxyurea, univariate COX regression analyses with baseline biological parameters were carried out after adjustment for ongoing hydroxyurea treatment at event and are presented in Table 3. Moreover, all multivariate analyses were also adjusted with ongoing hydroxyurea treatment.

**Table 3:** Predictive risk factors for intra- and extracranial arteriopathies in patients with SCD.

	**SCD patients (*****N*** = **493)**									
	**Intracranial TAMV**≥**200**		**Intracranial stenosis**		**Isolated eICA TAMV**≥**160**		**eICA kinkings**		**Isolated eICA Stenosis**	
	***N*** = **97/493**		***N*** = **35/375**		***N*** = **50/493**		***N*** = **100/365**		***N*** = **31/365**	
	**COX regression analysis after Adjustment with ongoing HU treatment at event Hazard Ratio (HR) and 95% Confidence Interval**									
	**HR (95% CI)**	***P*** **-value**	**HR (95% CI)**	***P*** **-value**	**HR (95% CI)**	***P*** **-value**	**HR (95% CI)**	***P*** **-value**	**HR (95% CI)**	***P*** **-value**
	**Per presence**									
**Genetic markers**										
Gender F vs. M		>0.2		>0.2		>0.2		>0.2		>0.2
G6PD deficiency	1.610 (0.960–2.703)	0.071	2.347 (1.048–5.236)	**0.038**		>0.2		>0.2		>0.2
Alpha-Thalassemia		>0.2	0.436 (0.190–1.002)	0.051		>0.2		>0.2		>0.2
	**Per beta-haplotype number**									
Beta Haplotype										
Car	1.415 (1.137–1.760)	**0.002**	1.351 (0.946–1.930)	0.098	1.280 (0.939–1.744)	0.118		>0.2		>0.2
Ben		>0.2		>0.2		>0.2		>0.2		>0.2
Sen	0.624 (0.423–0.921)	**0.017**		>0.2	1.311 (0.904–1.901)	0.154	1.330 (1.027–1.723)	**0.031**	1.579 (1.027–2.427)	**0.037**
	**Per unit increase**									
**Biological parameters**										
Hemoglobin level (g/dL)	0.196 (0.101–0.382)	**<0.001**	0.461 (0.336–0.633)	**<0.001**	0.683 (0.554–0.843)	**<0.001**	0.774 (0.671–0.894)	**<0.001**	0.721(0.551–0.945)	**0.018**
Hematocrit (%)	0.888 (0.863–0.914)	**<0.001**	0.871 (0.832–0.911)	**<0.001**	0.901 (0.851–0.953)	**<0.001**	0.925 (0.887–0.965)	**<0.001**	0.929 (0.862–1.002)	0.055
Reticulocyte count (10^9^/L)	1.006 (1.004–1.007)	**<0.001**	1.007 (1.004–1.009)	**<0.001**	1.002 (1.000–1.004)	0.077	1.002 (1.001–1.004)	**0.003**	1.002 (0.999–1.005)	0.126
WBC count (10^9^/L)	1.149 (1.111–1.187)	**<0.001**	1.145 (1.086—1.206)	**<0.001**	1.038 (0.984–1.095)	0.167		>0.2		>0.2
Neutrophil count (10^9^/L)	1.152 (1.090–1.217)	**<0.001**	1.119 (1.014-1.235)	**0.025**	1.077 (0.978–1.185)	0.131		>0.2		>0.2
Platelet count (10^9^/L)		>0.2		>0.2		0.198		>0.2		>0.2
MCV (fL)	1.069 (1.043–1.095)	**<0.001**	1.089 (1.043–1.137)	**<0.001**	1.030 (0.997–1.063)	0.072		>0.2		>0.2
Bilirubin (mmol/L)	1.030 (1.018–1.042)	**<0.001**		>0.2	1.015 (0.998-1.032)	0.079	1.011 (0.998–1.025)	0.085		>0.2
LDH (IU/L)	1.001 (1.001–1.002)	**<0.001**	1.002 (1.001–1.003)	**<0.001**		>0.2		>0.2		>0.2
HbF (%)		>0.2		>0.2		0.2	1.019 (0.995-1.043)	0.115		>0.2
**Thresholds**										
Hemoglobin <7g/dL	5.525 (3.247–9.346)	**<0.001**	2.278 (0.853-6.060)	0.100	2.247 (1.033–4.878)	**0.041**		>0.2		>0.2
WBC count > 20 x 10^9^/L	5.236 (3.115–8.772)	**<0.001**	3.363 (1.558-8.621)	**0.003**		>0.2		>0.2		>0.2
Reticulocyte count > 400 x 10^9^/L	3.817 (2.299–6.329)	**<0.001**	3.559 (1.541–8.264)	**0.003**		>0.2		>0.2		>0.2

Univariate Cox regression analysis after adjustment with ongoing hydroxyurea treatment at each event. Levels of thresholds used were the 10th percentile for hemoglobin (7.0 g/dL) and near the 90th percentile for WBC (19.7 x 10^9^/L) and reticulocyte counts (422 x 10^9^/L) but rounded for easier clinicaluse.

The bold values indicates the significant *P*-values (<0.05).

### Intracranial arteriopathy

#### Abnormal high intracranial TAMVs ≥200 cm/s

Multivariate Cox regression analysis introducing all risk factors <0.2 presented in Table 3 (except hematocrit and neutrophil counts strongly correlated with hemoglobin and WBC counts, respectively) retained as significant and independent predictive risk factors: baseline hemoglobin level, which, per unit increase, decreased the risk [HR = 0.495 (0.401–0.613), *p* < 0.001], and the WBC count, which increased the risk [HR = 1.098 (1.055–1.143), *p* < 0.001], independently of ongoing hydroxyurea treatment. For the model predicting abnormal high intracranial TAMV including hemoglobin and WBC, the P-value for the proportional hazard (PH) assumption test was *P* = 0.84, indicating no violation of the PH assumption.

Using thresholds, the analysis retained baseline hemoglobin <7g/dL [HR = 4.630 (2.703–7.937), *p* < 0.001], WBC >20x10 (9)/L [HR = 3.774 (1.876-6.494), *p* < 0.001], and reticulocytes >400 x 10^9^/L [HR = 2.247 (1.321–3.817), *p* = 0.003] as significant and independent predictive risk factors.

#### Intracranial stenosis

Multivariate analysis retained only as predictive risk factors: baseline hemoglobin [HR = 0.537 (0.382–0.755), *p* < 0.001], which decreased the risk, and WBC count [HR = 1.098 (1.033–1.168), *p* = 0.003], which increased the risk of intracranial stenosis occurrence independently of ongoing hydroxyurea treatment.

### Extracranial arteriopathy

#### Isolated eICA-TAMVs ≥160 cm/s

Multivariate Cox regression analysis retained as independent predictive risk factors: baseline hemoglobin [HR = 0.739 (95% CI: 0.583–0.937), *p* = 0.013] and SEN beta-haplotype number [HR = 1.653 (95% CI: 1.064–2.566), *p* = 0.025], which per unit increase decreased and increased the risk of eICA-TAMVs ≥160 cm/s, respectively. For the model predicting abnormal high eICA-TAMVs including hemoglobin and SEN beta-haplotype number, the P-value for the PH assumption test was *P* = 0.96, indicating no violation of the PH assumption.

#### eICA kinkings

Multivariate Cox regression analysis retained as significant and independent predictive risk factors for eICA kinkings: baseline hemoglobin [HR = 0.757 (95% CI: 0.627–0.915), *p* = 0.004], which, per unit increase, decreased the risk and SEN beta-haplotype number [HR = 1.453 (95% CI: 1.108–1.905), *p* = 0.007], which, per unit increase, increased the risk, independently of ongoing hydroxyurea treatment.

Of note, the prevalence of kinkings in SCD patients without SEN beta-haplotype was 29.2% (66/226) vs. 35.1% (13/37) in those with one and 45.9% (17/37) in those with two SEN beta-haplotypes.

#### eICA stenosis

Multivariate Cox regression analysis retained as significant and independent predictive risk factors for eICA stenosis: baseline hemoglobin [HR = 0.681 (95% CI: 0.506–0.917), *p* = 0.011] and SEN beta-haplotype number [HR = 1.697 (95% CI: 1.096–2.626), *p* = 0.018], respectively, independently of ongoing hydroxyurea treatment.

## Impact of the SEN beta-haplotype

**Figure 5:**
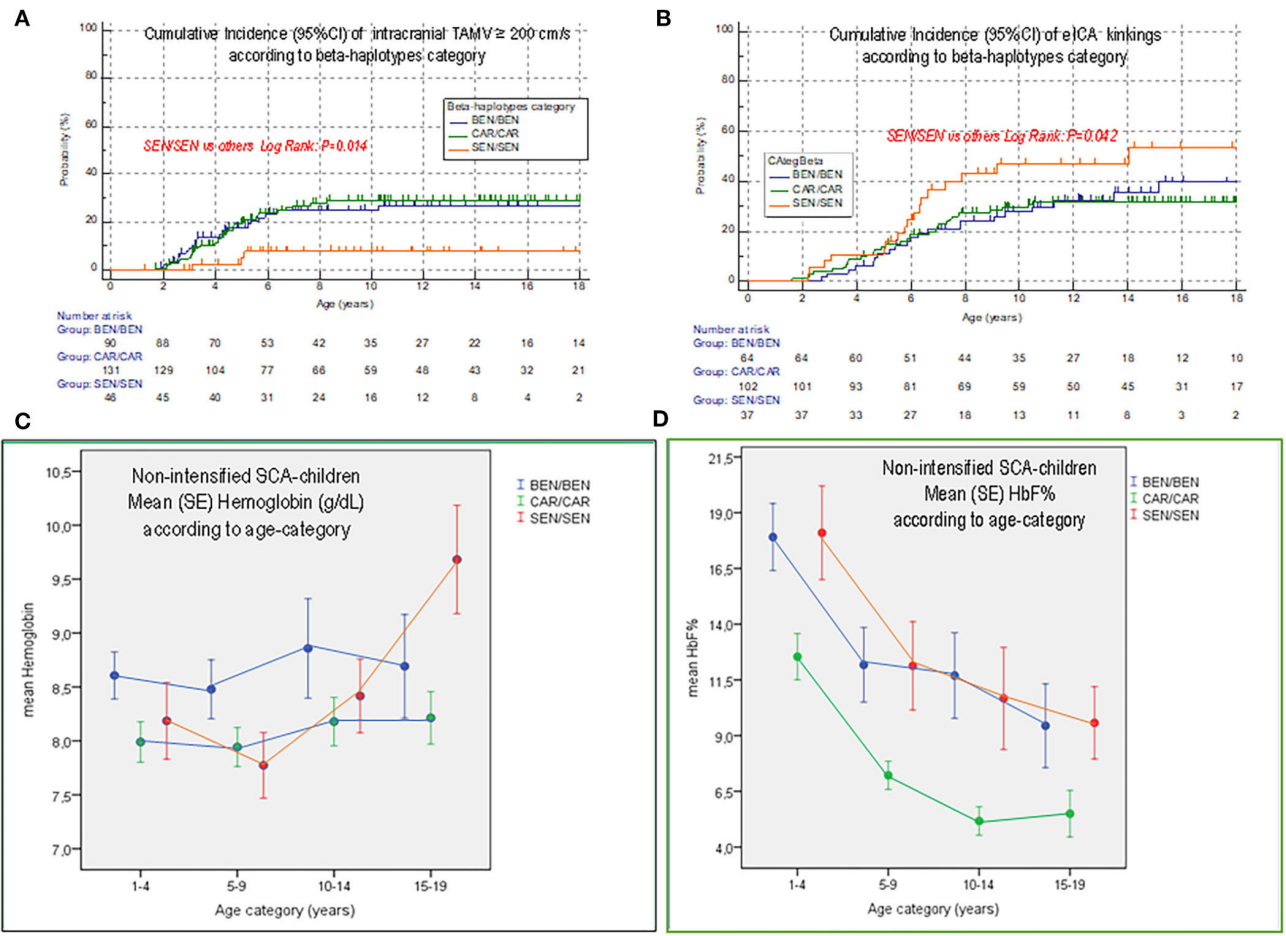
Comparison of cumulative incidence of intra- and extracranial TAMV and kinkings according to homozygous beta-haplotype category and outcome of hemoglobin level and HbF% in non-intensified children according to the three beta-haplotypes categories. **(A)** Cumulative incidence of intracranial TAMV ≥ 200 cm/s according to homozygous beta-haplotypes category. **(B)** Cumulative incidence of eICA kinkings according to homozygous beta-haplotypes category. **(C)** Outcome of hemoglobin level during aging in non-intensified patients (not on hydroxyurea, or on chronic transfusion, or transplanted) according to homozygous beta-haplotypes category. **(D)** Outcome of HbF% level during aging in non-intensified patients according to homozygous beta-haplotypes category. These values were recorded at annual checkup in non-intensified children with SCA. While mean hemoglobin remained relatively stable during aging in children with BEN/BEN and CAR/CAR, hemoglobin decreased in children with SEN/SEN between 5 and 10 years of age before significantly increasing thereafter. Thus, children with SEN/SEN were the most anemic between 5 and 10 years of age and the less anemic after 15 years of age. This age period corresponds with the occurrence of abnormal TAMV and eICA kinkings in patients with SEN/SEN.

While the presence of SEN beta-haplotypes was protective for the development of intracranial TAMVs ≥200 cm/s, it was a risk factor for eICA-TAMVs ≥160 cm/s and eICA kinkings, with a maximum impact in the presence of two SEN beta-haplotypes (Figures 5A,B). As children with SEN/SEN have the highest baseline hemoglobin compared to other beta-haplotypes, the lowest probability of intracranial arteriopathy was not surprising, but the highest risk of extracranial arteriopathy was unexpected. As the mean (SD) age at hydroxyurea initiation in SEN/SEN [6.2 yr, (3.2)] was slightly higher than that in BEN/BEN [5.2 yr, (2.8)] and CAR/CAR [5.4 yr, (2.9)] children, although not significantly, we determined the changes in hemoglobin and HbF% with aging in the non-intensified patients (no hydroxyurea, chronic transfusion, or transplantation). We observed that in children with SEN/SEN, but not in children with other haplotypes, hemoglobin strongly decreased between 5 and 10 years of age, a period during which they developed intracranial TAMVs ≥200 cm/s and eICA kinkings (Figures 5C,D).

## Discussion

Here, we present the first longitudinal cohort study including 493 children with SCD simultaneously assessed by transcranial and cervical color Doppler ultrasound. In the present SCD cohort study, only children with SCA and not SC/Sβ+children developed both intra- and extracranial macro-arteriopathies during infancy. Lower intracranial TAMVs in patients with SC were previously reported (23), but the cumulative incidence of cerebral arteriopathy in longitudinal cohort studies has been missing in the literature, and the benefit of TCD screening in children with compound heterozygous SCD is not well defined (24). The present study showing the absence of cerebral macro-arteriopathy in SC/Sβ+ children raises the issue of the usefulness of early TCD screening in this population. However, as kinkings were observed in several SC/Sβ+ children in this cohort, and SCI has been reported in this population, (25, 26) the data suggest that the presence of eICA stenosis should be investigated in adult patients.

In SCA, most ischemic strokes are linked to intracranial arteriopathy, but the circle of Willis is normal in approximately 25% of cases (9), indicating an additional underlying mechanism. Interestingly, several cases of strokes have been reported to be associated with stenosis/occlusion of eICA (1, 10–13). Moreover, the incidence of SCI was significantly associated with the presence of eICA stenosis (7). Until now, cerebral MRA and TCD have been mainly used to explore the major intracranial vessels. Compared with those of the intracranial vasculature, morphologic changes of the extracranial vasculature in patients with SCD are less well described and understood. Cervical color Doppler ultrasound scanning has only recently been used in SCD. Using a submandibular approach, Gorman et al. (11) detected abnormal eICA-TAMVs in four of 131 children, three of whom had a stroke, while 13 of 236 children had eICA stenosis or occlusion in Deane et al.'s study (12). Telfer et al. used neck MRA in 67 SCA patients with a history of stroke or abnormal TCD and found that 10 of 67 (15%) had eICA occlusion (13). We reported a cross-sectional study between June 2011 and April 2012 in two stroke-free cohorts including 435 SCA cases assessed by transcranial and cervical Doppler, with intracranial and neck MRA available in 104 of 435 subjects (14). We showed that TAMVs were about 25% lower in the eICA than in the MCA and that eICA-TAMVs ≥160 cm/s were highly associated with eICA stenosis and kinkings.

The present longitudinal cohort study is the first to show that the kinetics of extracranial arteriopathy in patients with SCA are similar to those of intracranial arteriopathy as abnormal eICA-TAMV and stenosis were observed as soon as the 2nd year of life, in line with Telfer's study reporting the occurrence of four stroke episodes associated with eICA occlusion in young children at 2 to 4 years of age (13). Importantly, we show that the cumulative incidence of abnormal eICA-TAMV reaches a plateau by about 10 years of age, as previously shown for intracranial abnormal TAMV in children with SCA (17). The importance of systematically detecting extracranial arteriopathy is supported by the ability of eICA assessment to identify 13.5% additional patients at risk of stroke because of eICA-TAMVs ≥200 cm/s or eICA stenosis in the absence of intracranial arteriopathy. A cross-sectional study in 167 children and adults with SCA assessed by cerebral MRI/MRA and neck MRA reported the presence of intra- or extracranial stenosis in 20 and nine patients, respectively, but extracranial stenosis was always associated with the presence of intracranial stenoses and could not be evaluated as an independent risk factor for stroke (27). Thus, the authors suggested that systematic detection of extracranial stenosis may have limited clinical utility in patients with SCD (27). However, Verlhac et al. (28) recently reported in the Debré, Paris, cohort the presence of eICA stenosis in 48 children with SCA, of whom eight had a history of stroke; importantly, five of eight had isolated eICA stenosis, confirming the clinical utility to systematically assess the extracranial part of the internal carotid artery.

We also show for the first time that in addition to a congenital origin, eICA kinkings in patients with SCD can develop progressively with aging as a function of eICA-TAMVs, themselves related to anemia severity. Kinkings are known to be increased in a variety of connective tissue genetic disorders, such as Loeys–Dietz syndrome (29). They are associated with a risk of eICA dissection (30) and transient cerebral arteriopathy and may represent a clinically relevant imaging biomarker of vascular biology for pediatric strokes (31). The congenital or acquired origin of kinkings is still debated (32). Their non-association with age has been reported, suggesting that they are the result of alterations during embryological development, rather than vascular remodeling secondary to aging (33). In SCA children, they may promote the formation of stenosis by disturbing blood flow, for example, in the intracranial carotid siphon and bifurcation. However, other studies have described predisposing factors such as older age, female gender, and hypertension, suggesting vascular remodeling. A study compared the presence of eICA kinkings assessed by neck MRA in 56 patients with SCD with 56 controls and showed significantly greater tortuosity in the eICA and vertebral arteries in patients with SCD than in controls, suggesting that they could be due to aberrations in hemodynamics from nonlaminar flow in these vessels (34). Our findings showing progressive development of eICA kinkings during infancy argues in favor of vascular remodeling as a consequence of high blood flow associated with severe chronic anemia. This is also supported by studies using an arteriovenous fistula model in rabbits, showing that high blood flow and high shear stress [defined as the tangential force per unit area exerted by the wall on the fluid (35)] induced endothelial cell activation, proliferation, (36) dilatation, elongation, and tortuosity, and smooth muscle cell proliferation (37). When arteries are enlarged and remodeled in response to high flow conditions, wall shear stress is subsequently reduced, and long-term exposure to low wall shear stress (<5 dynes/cm^2^) induces severe intimal thickening (37). Worsening of eICA tortuosity can induce a blood pressure drop (38) responsible for cerebral ischemia occurrence when self-regulatory mechanisms cannot compensate for the blood flow drop. This could explain the association between eICA stenosis and the presence of SCI (7). Nevertheless, additional genetic factors promoting eICA kinkings cannot be excluded, as suggested by the increased incidence of kinkings and extracranial arteriopathy observed in this series in patients with SEN beta-haplotype.

While the SCA genotype and low baseline hemoglobin level were predictive risk factors for intra- and extracranial arteriopathies, high baseline reticulocyte count and LDH, the surrogate markers for hemolysis, and WBC count were only predictive of intracranial arteriopathy risk. SEN-β-haplotypes which were found associated with a lower risk for intracranial-arteriopathy, were unexpectedly associated with higher risk for extracranial arteriopathy, but showed in non-intensified patients that SEN/SEN children had decreased hemoglobin between 5 and 10 years of age, the period when they start developing eICA-kinkings with higher frequency compared to other beta-haplotypes. Another explanation could be a genetic link between the ethnic origin from Senegal and the presence of kinkings. Thus, it would be important to compare the prevalence of kinkings in non-SCD patients from Senegal with other African countries. This finding could explain the higher incidence of SCI observed in patients with the SEN β-haplotype (39), as we previously showed an association between eICA kinkings and SCI (7).

Could our findings impact the choice of therapy for cerebral arteriopathy prevention? The present prospective cohort study was not designed to test the impact of hydroxyurea treatment, which was only given in patients with normal cerebral velocities or in those with a history of abnormal cerebral velocities who had normalized them on chronic transfusion and had no arterial stenosis. However, we show that ongoing hydroxyurea treatment for at least 6 months at event was associated with a lower risk of intracranial arteriopathy, and we show, for the first time, an association with a lower risk of eICA kinking development. As severe baseline anemia is the major predictive risk factor for intra-/extracranial arteriopathy and SCI (7, 40), it is clear that any treatment improving anemia such as hydroxyurea or voxelotor (41) could be useful to decrease the risk of intra-/extracranial arteriopathy. Hydroxyurea induces the increase in HbF, MCV, and hemoglobin; decreases WBC, neutrophil, and platelet counts; improves hemolytic markers [decreased reticulocyte count and LDH (42)]; and significantly decreases the rate of VOC, ACS, and transfusion needs (43). The safety and efficacy of hydroxyurea in young children have been established in the United States (22) and in malaria-endemic sub-Saharan Africa (44, 45), justifying early hydroxyurea initiation, not only to prevent crises but also to secondary strokes in low-income countries where safe blood products are rare and costly. As hemolytic anemia and high WBC count are the major risk factors for intracranial arteriopathy, hydroxyurea is a good candidate for primary stroke prevention. Open-label trials in the United States and Nigeria (46–51), and several randomized trials comparing hydroxyurea with the placebo such as BABY HUG (21, 52) (the United States), SCATE (53) (the United States, Jamaica, and Brazil), and NOHARM (54) (Uganda) have shown that hydroxyurea lowers velocities in patients with elevated intracranial velocities and reduces the risk of conversion to abnormal velocities (46–51) and of first clinical strokes. Thus, to date, there are robust data showing that hydroxyurea decreases the risk of abnormal intracranial TAMVs. In France, providers were initially reluctant to systematically treat asymptomatic young children with hydroxyurea due to the cytotoxic effect of hydroxyurea on dividing cells, including spermatogonia (55–57). However, a recent report in patients treated with hydroxyurea prior to puberty, whose sperm parameters were analyzed after treatment suspension and several months on chronic transfusion, showed no specific effect of hydroxyurea on sperm parameters above those SCD-related (58), supporting its early use. Nevertheless, in the absence of systematic cerebral MRI/MRA and neck MRA in those studies comparing hydroxyurea with placebo, the impact of hydroxyurea on decreasing the risk of stenosis, SCI, and extracranial arteriopathy remains unknown. The HUSTLE study, evaluating the prevalence of SCI in a cohort treated with hydroxyurea at maximum tolerated dose, found only a small increase in the prevalence of SCI on hydroxyurea (38% at baseline and 41% after 6 years) (59). By contrast, an increase in detection and progression of SCI despite hydroxyurea was reported in another study (60). Our data showing a higher risk of extracranial arteriopathy in patients with SEN β-haplotype who have a higher baseline HbF level do not argue for a favorable impact of hydroxyurea on extracranial arteriopathy. It would be important to evaluate in independent cohorts with a high prevalence of patients with SEN β-haplotype if early initiation of hydroxyurea before age 2 years could prevent the strong decrease in hemoglobin observed in our cohort among SEN/SEN patients between 5 and 10 years of age and the extracranial arteriopathy. However, we have reported that compared to children with SEN/SEN, children with BEN/BEN and CAR/CAR had the highest hemoglobin and HbF levels at baseline, and they had significantly lower hemoglobin and HbF levels on hydroxyurea than children with BEN/BEN who had the best response to hydroxyurea (61). Other factors may explain the variable impact of hydroxyurea on cerebral vasculopathy. The increased affinity of HbF for oxygen can decrease tissue oxygen delivery and explain the variable efficiency of hydroxyurea in preventing SCI, priapism, and pulmonary hypertension (62). Moreover, the increased MCV observed on hydroxyurea could be deleterious in the absence of sufficient improvement of deformability. Thus, in the presence of severe cerebral arteriopathy, chronic transfusion appears more efficient than hydroxyurea to reduce stroke recurrence, as demonstrated in the SWiTCH trial (63), and transplantation, which can cure 98% of children with SCA (64), and reduces TAMVs (65) and stenosis scores (66) more efficiently than chronic transfusion, as demonstrated in the DREPAGREFFE trial (65). Finally, gene therapy using lentiviral transfer of a marked β-globin (β^A−T87Q^) gene (67), which results in near pancellular β^A−T87Q^ expression with reduced sickling and hemolysis and increased total hemoglobin (68), seems quite promising for cerebral vasculopathy prevention.

This study has some limitations. The relatively low number of SC/Sβ+ children in this series does not allow to conclude that this population never develops extracranial arteriopathy during infancy. For several outcomes, including intracranial TAMVs ≥ 200 cm/s, intracranial stenosis, eICA TAMVs ≥ 160 cm/s, and eICA stenosis, the number of events in the patients with SC/Sβ+ was either 0 or very small, and it was therefore not possible to accurately calculate hazard ratios. It was not designed to evaluate the impact of hydroxyurea on cerebral vasculopathy prevention as hydroxyurea was only given to children with SCA with normal TAMVs and no stenosis. We had a high proportion of patients receiving chronic transfusion, and our results may not be generalizable to cohorts with lower rates of transfusion. Moreover, the associations between biological parameters and cerebral arteriopathy described here are only descriptive and do not demonstrate any causal relationship. We also acknowledge that our results will need to be further validated in independent cohorts with a sufficient proportion of patients with SEN β haplotypes and homogenously treated early with hydroxyurea. However, this is the first prospective longitudinal cohort study reporting the kinetics and predictive risk factors for extracranial arteriopathy in children with SCD followed in the same referral center since birth, and highlighting the predictive and critical values of baseline parameters recorded during the 2nd year of life before any intensive therapy. These blood parameters are representative of the influence of all genetic biomarkers on the different blood components. Thus, children with baseline hemoglobin <7g/L, WBC count >20 x 10^9^/L, or reticulocyte >400 x 10^9^/L should be considered as candidates for early intensified therapy, transplantation in the presence of matched sibling donor, or gene therapy.

In conclusion, only children with SCA appear to be at risk of intra- and extracranial arteriopathies during infancy. Extracranial arteriopathy is most often isolated, and eICA assessment detects 13.5% more patients at risk of stroke not detected by intracranial assessment, showing the importance of systematically assessing the eICA in children with SCA. In addition to a congenital origin, eICA kinkings in patients with SCD can develop progressively with aging as a function of eICA-TAMVs, themselves related to anemia severity, and are risk factors for stenotic extracranial arteriopathy. This prospective cohort study shows the importance of recording biological parameters during the 2nd year of life before any intensive therapy to predict the risk of cerebral arteriopathy and treat patients with severe baseline anemia.

## Data availability statement

The original contributions presented in the study are included in the article/supplementary material, further inquiries can be directed to the corresponding author/s.

## Ethics statement

The studies involving human participants were reviewed and approved by Créteil-Institutional-Review-Board. Written informed consent to participate in this study was provided by the participants' legal guardian/next of kin.

## Author contributions

FB designed and performed the research, collected the data, performed the statistical analyses, interpreted the data, and wrote the manuscript. SV designed and performed the research, performed Doppler ultrasound scans and MRI/MRA, collected, analyzed, and interpreted data, and co-wrote the manuscript. CA, AK, and CJ designed the study, collected and interpreted data, and co-wrote the manuscript. FB, CA, AK, IH, FM, and RE participated in the management of patient care. All authors critically reviewed and approved the manuscript.

## Conflict of interest

The authors declare that the research was conducted in the absence of any commercial or financial relationships that could be construed as a potential conflict of interest.

## Publisher's note

All claims expressed in this article are solely those of the authors and do not necessarily represent those of their affiliated organizations, or those of the publisher, the editors and the reviewers. Any product that may be evaluated in this article, or claim that may be made by its manufacturer, is not guaranteed or endorsed by the publisher.
